# Completion of Metal-Damaged Traces Based on Deep Learning in Sinogram Domain for Metal Artifacts Reduction in CT Images

**DOI:** 10.3390/s21248164

**Published:** 2021-12-07

**Authors:** Linlin Zhu, Yu Han, Xiaoqi Xi, Lei Li, Bin Yan

**Affiliations:** Henan Key Laboratory of Imaging and Intelligent Processing, PLA Strategic Support Force Information Engineering University, Zhengzhou 450001, China; zhulinlingfs@163.com (L.Z.); hy007hy007@126.com (Y.H.); XqXi2021@163.com (X.X.); leehotline@aliyun.com (L.L.)

**Keywords:** computed tomography, metal artifacts reduction, deep learning, sinogram

## Abstract

In computed tomography (CT) images, the presence of metal artifacts leads to contaminated object structures. Theoretically, eliminating metal artifacts in the sinogram domain can correct projection deviation and provide reconstructed images that are more real. Contemporary methods that use deep networks for completing metal-damaged sinogram data are limited to discontinuity at the boundaries of traces, which, however, lead to secondary artifacts. This study modifies the traditional U-net and adds two sinogram feature losses of projection images—namely, continuity and consistency of projection data at each angle, improving the accuracy of the complemented sinogram data. Masking the metal traces also ensures the stability and reliability of the unaffected data during metal artifacts reduction. The projection and reconstruction results and various evaluation metrics reveal that the proposed method can accurately repair missing data and reduce metal artifacts in reconstructed CT images.

## 1. Introduction

Computed tomography (CT) is one of the primary methods of nondestructive testing, and it is widely used in many fields such as medicine [1] and industry [2]. Different materials have different attenuation coefficients for X-rays, because of which, we obtain projection data with information about materials inside an object. When the scanned object contains a high-density structure such as metal, because of the strong attenuation of the metal, the X-rays cannot fully penetrate the object, which causes a dark shadow in the measured sinogram [3]. These values no longer satisfy Beer’s law, resulting in information loss in reconstructed CT images because of the presence of metal artifacts. Metal artifacts are diverse and spread across an image; they severely degrade image quality and interfere with subsequent analysis and processing. After more than 40 years of research, there is still no general solution for metal artifacts reduction (MAR). MAR is still a common and challenging problem in CT research [4].

Contemporary methods of MAR mainly include iteration reconstruction and projection interpolation. Iterative reconstruction methods typically use a model-based approach to minimize well-defined objective functions. In 1996, Wang et al. [5] proposed the algebraic reconstruction technique (ART), a deblurring technique, for MAR. Xue et al. [6] used a total variation iteration algorithm to patch metal projections. Wang et al. [7,8] used the penalized weighted least-squares algorithm to iteratively reconstruct the position and shape of metal objects in CT images. Zhang et al. [9] proposed a MAR method for weighted total variational constraint reconstruction by analyzing how the absence of projection information is affected by pixel positions around a metallic object. The algorithm could considerably suppress metal artifacts and noise; however, metal region information was lost in the correction reconstruction results. Choi et al. [10] proposed a sparsity-driven iterative reconstruction algorithm for obtaining information of metallic regions after MAR. The algorithm could accurately determine the location and shape of a metal object in an image; however, the correction results presented secondary artifacts. Levakhina et al. [11] proposed a simultaneous iterative reconstruction algorithm to correct metal artifacts by introducing a weight factor in back projection for limiting the contribution of projection values to the reconstruction across metal voxels; this could more effectively suppress artifacts owing to the high density of metallic objects.

In 1987, Kalender et al. [12] treated metal-damaged data as missing data and estimated them using a one-dimensional linear interpolation method (LI-MAR). Mahnken et al. [13] proposed a two-dimensional interpolation method that replaces metal-damaged data with a weighted sum of 16 nearest neighbors. Bal et al. [14] first proposed using the mean clustering method to calculate a prior model. This algorithm could effectively protect the high-density structure of the metal edges; however, the serious issue of secondary artifacts remained. Meyer et al. [15] proposed the normalized MAR (NMAR) algorithm using a priori model for normalized interpolation. The NMAR algorithm could more effectively suppress secondary artifacts and protect the structures of metal edges. Jeon S. et al. [16] used forward projection of a modified CT image and generated a sinogram containing information about the structure of the metallic object.

In recent years, deep learning has rapidly gained attention in various fields, especially image processing [17,18,19]. Deep learning has been successfully applied in image restoration and denoising, providing new methods for MAR in CT images [20]. Gjesteby et al. [21] first introduced CNN in MAR in the image domain. Zhang et al. [22] proposed combining the results obtained using different methods as a prior image to improve the effect of artifact correction. In another study [23], Gjesteby et al. trained NMAR and neural network fusion using the results of NMAR, and the detailed image extracted by the guided filter was given as hybrid input to a dual-stream network. Liao et al. [24] proposed a novel artifact disentanglement network that enabled different forms of generations and regularizations between the artifact-affected and artifact-free image domains to support unsupervised learning. Claus et al. [25] proposed a MAR method to enable the interpolation of missing data in the sinogram domain by training a fully connected neural network (FCN). Park et al. [26] used U-net [27] to repair inconsistent sinograms by removing the primary metal-induced beam hardening factors along the metal trace boundaries. In [28,29,30], generative adversarial net (GAN) and its variants were introduced to improve the in-painting performance. Partial convolution [31] was employed in [32,33] for sinogram completion. In the sinogram domain, MAR can correct projection deviation and provide more realistic reconstructed images. However, ensuring sinogram continuity at the boundaries of metal-damaged traces is challenging, and secondary artifacts are inevitable.

In the sinogram domain, only the projection values are affected by the presence of metallic objects and change in a nonlinear manner. MAR, in the sinogram domain, can maximize the use of the original effective projection information. Preliminary studies have applied deep learning methods for sinogram data completion; however, these are limited to smaller metal implants and highly constrained scenarios. In this study, deep learning was applied to traditional U-net layers to complete metal-damaged traces, and then filtered back projection (FBP) was used to reconstruct a complete image. The consistency of projection data at each angle and the continuity of projection data were designed as sinogram feature losses, and masking was used so that the network only complements the metal-damaged data, ensuring stability and reliability of the unaffected data. The network was trained using a large number of training samples.

## 2. Materials and Methods

**Figure 1:**
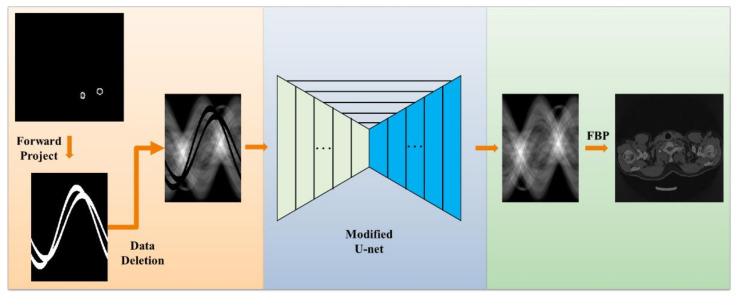
Workflow and major components of the proposed MAR framework.

The workflow of our method is illustrated in Figure 1. First, training data and simulation were generated, followed by the removal of metal-damaged projection data. We used sinogram data in a scene that did not contain metal objects and then created a matching sinogram by deleting data corresponding to the geometric configuration of that metal object; next, the network was designed and trained, that is, U-net based sinogram completion. We modified the original U-net to improve the data filling performance and the generalization ability of the model; lastly, FBP images were reconstructed efficiently.

### 2.1. Dataset Generation

The primary impact of metal artifacts in the sinogram is the nonlinear change in the metal-damaged trace values; other effects are relatively weak. The presence of a metallic object did not affect our method because we mainly carried out the completion of missing data. Compared with the scanned object, we designed a phantom containing only metal. The phantom was scanned using a simulated X-ray scanner to obtain a sinogram, which was binarized and used as the metal mask. The values corresponding to the metal projection position in the projection sinogram were set to zero. Thus, we manually generated a large number of real pairs of incomplete and complete sinograms to train our network. In the process of generating the dataset, this study used sinogram images with a size of 512 × 360. During sample production, only the metal-damaged traces were set to zero, ensuring the stability and validity of other projection values.

In the testing process, for the metal present in the actual scan, we carved out the metal trace area in the sinogram. First, we performed reconstruction and used the threshold segmentation method on the reconstructed image to obtain metal location and size, and then, we used reprojection to obtain the corresponding projected traces. To ensure complete identification of the metal trace area, the metal size could be expanded as required.

### 2.2. Network Architecture

In recent years, advances in deep neural networks have provided new ideas for MAR in CT images. According to the general approximation theorem [34], a projection can be completed using a multilayer neural network. In this study, the original U-net was modified to optimize the data filling performance and generalization ability of the model.

**Figure 2:**
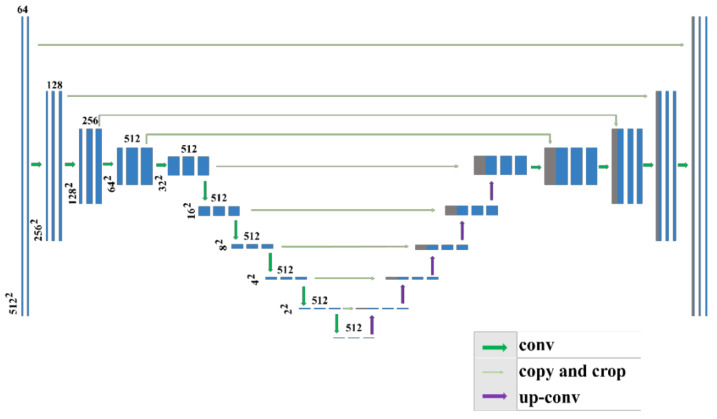
Modified U-net architecture.

Figure 2 shows the modified U-net structure, which mainly included three parts—namely, an encoding module, a decoding module, and a skip connection. The network mapped the depth features, extracted the feature maps through multilevel nonlinear mapping, and then fused the feature maps to complete the sinogram. The encoding module was mainly used to extract feature information from the input sinogram domain images. This module contained 10 dimensionality reduction blocks; each block contained two convolution operations with a convolution kernel size of 3 × 3 and stride size of 1, and one convolution demodulation operation with a convolution kernel size of 3 × 3 and stride size of 2. In the first three dimensionality reduction blocks, the number of convolution kernel channels was 64, 128, and 256, respectively, and from the fourth to the tenth layer, it was 512. For all convolutional layers, the activation function was a rectified linear unit (ReLU) with a slope of 0.2. To ensure optimal use of the feature correlation between the image pixels, we removed the pooling layer in the original network and used a convolution operation with a stride size of 2, to complete the dimension reduction.

The decoding module comprised 10 corresponding ascending blocks and deconvolution with a stride size of 2. The convolution kernel of the first six deconvolution blocks was 3 × 3, and the number of convolution channels was 512. The convolution kernel from the eighth layer to the tenth layer was 3 × 3, and the number of convolution channels was 256, 128, and 64, respectively. The activation function of these blocks was ReLU.

The skip connection connected the corresponding encoding and decoding modules. In this network, the output and input images were of the same size. To maintain the size of the image, a padding operation was added. Each slice in the CT image of the dataset considered in this study was a single channel; therefore, the input image size of the network was changed from 224 × 224 × 3 to 512 × 512 × 1.

### 2.3. Loss Function and Training

**Figure 3:**
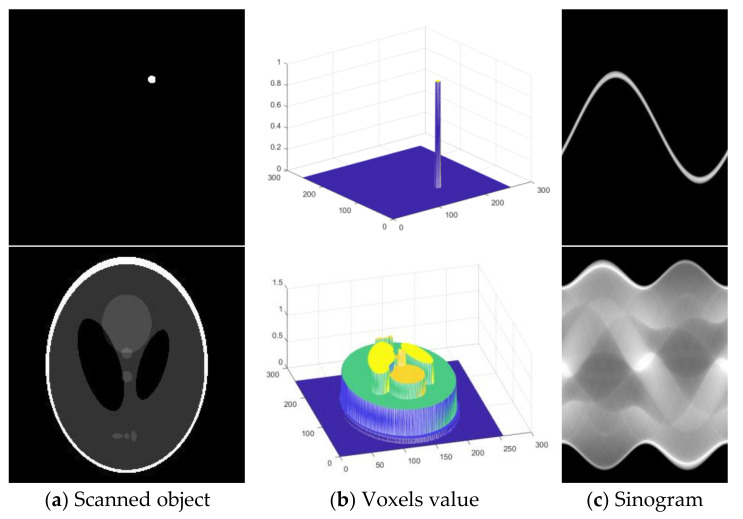
Schematic diagram of data characteristics of a projection sinogram.

The data characteristics of projection in CT imaging are shown in Figure 3. A single voxel distribution trajectory in a projection space is a sine function of the scanning angle of the scanned object. A sinogram is the sum of the projected sinusoids of all voxels, and it has high-order continuity similar to the sine function. The increasing complexity of internal structure information leads to the increasing complexity of the sinogram. It is challenging to construct a traditional mathematical model that can accurately describe the data characteristics of a sinogram.

A CT image reflects the attenuation coefficients of a scanned object. In the scanned object, the change in the attenuation coefficient is continuous, especially in medical CT examinations. Sinogram data are the integral of the attenuation coefficients of a scanned object. Integral transformation makes the image more continuous and strengthens local correlation. Therefore, CT projection data have more robust local correlations [35].

**Figure 4:**
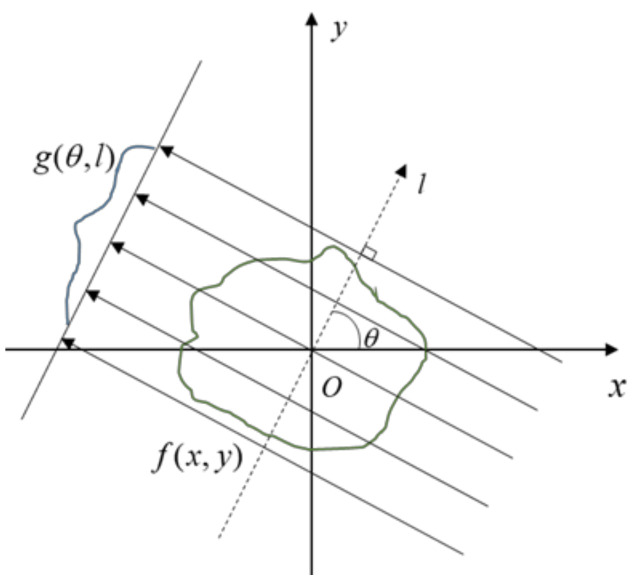
Parallel beam geometry of CT.

Helgason–Ludwig consistency conditions (HLCCs) are widely used in CT image artifact correction and sinogram data recovery [36]. A parallel beam of X-rays at a specific angle is selected for the theoretical derivation of HLCC, as shown in Figure 4, where θ is the projection angle of the parallel X-rays, f(x,y) represents the scanned object in a two-dimensional space, and its projection under θ is g(θ,l). The k-order momentum of g(θ,l) is defined as

(1) $$V_{k}(\theta )=\int_{-\infty }^{\infty }l^{k}g(\theta ,l)dlk\geq 0$$

The image geometric momentum is defined as

(2) $$m_{i,j}=\iint x^{i}y^{j}f(x,y)dxdy$$

When $M_{k}(\theta )=\sum_{r=0}^{k}\left(\begin{array}{c} k\\ r \end{array}\right)m_{r,k-r}\cos^{r}\theta \sin^{k-r}\theta$, the k-order momentum of the projection $V_{k}(\theta )$ and the geometric momentum of the image $m_{i,j}$ satisfy Equation (3).

(3) $$V_{k}(\theta )=M_{k}(\theta )$$

Equation (3) is the HLCC equation, which satisfies i+j=k. When i=j=0, we obtain

(4) $$m_{0,0}=\iint f(x,y)dxdy=V_{0}(\theta )=\int_{-\infty }^{\infty }g(\theta ,l)dl$$

The HLCC equation shows that the sum of the line integrals of 2D parallel projection data at any projection angle is a constant independent projection angle under parallel beam geometry architecture.

In the process of three-dimensional cone-beam projection, the HLCC condition is satisfied under a small beam–cone angle. The integral sum of projections under a single angle is constant. When the scanned image contains metallic objects, because all angles are different, there are nonlinear changes in the projection result in the integral sums.

The proposed method limits the completion area to the metal-damaged traces, ensuring that the output image is similar to the input image. We used the L2 function as the global loss. The L2 loss has a more severe penalty for errors and can prevent significant deviations during the entire process, ensuring the reliability of the processed image. When the metal-damaged trace was set to zero, the edge of the metal trace exhibited considerable numerical change. Existing methods have limits, as they cannot ensure the continuity of the sinogram of the metal trace boundary. Amplitude loss was introduced to ensure continuity of the processed image. Difference loss was introduced so that the completed data satisfy the HLCC. The formulas for each type of loss are given as follows:

(5) $$L2_{loss}={\sum_{x=1}^{512}\sum_{\theta =1}^{360}\left[M⊗f\left(x,\theta \right)-M⊗\overset{\wedge }{f}\left(x,\theta \right)\right]}^{2}$$

(6) $$Amp_{loss}={\sum_{x=1}^{511}\sum_{\theta =1}^{360}\left[\left[f\left(x+1,\theta \right)-f\left(x,\theta \right)\right]-\left[\overset{\wedge }{f}\left(x+1,\theta \right)-\overset{\wedge }{f}\left(x,\theta \right)\right]\right]}^{2}$$

(7) $$Diff_{loss}={\sum_{\theta =1}^{360}\left[\sum_{x=1}^{512}f\left(x,\theta \right)-\sum_{x=1}^{512}\overset{\wedge }{f}\left(x,\theta \right)\right]}^{2}$$

(8) $$Loss=L2_{loss}+Amp_{loss}+Diff_{loss}$$

where f and $\overset{\wedge }{f}$ represent the network output sinogram and label sinogram, respectively, M is the metal mask, and ⊗ denotes element-wise multiplication. This operation focuses only on metal-damaged traces. x indicates the position of the detector element position, and θ represents the projection angle.

We trained and tested the network on Tensor-Flow (version 1.4.0) (Google, USA) on an AMAX workstation with two Intel Xeon E5-2630 v4 CPU (Intel, USA) 2.4 GHz and 64 GB memory and a GeForce GTX 1080Ti GPU (NVIDIA Corporation, USA) with 11 GB memory.

## 3. Results

### 3.1. Evaluation Metrics

In the sinogram domain, mean absolute error (MAE) was introduced as an evaluation metric to quantify the quality of the processed images. In the image domain, root-mean-square error (RMSE) and normalized mean absolute distance (NMAD) were introduced as the quality evaluation metrics for the reconstructed image. The formulas for calculating MAE, RMSE, and NMAD are as follows:

(9) $$\mathrm{MAE}=\left(\sum_{i=1}^{N}\left|\overset{\wedge }{f}\left(i\right)-f\left(i\right)\right|\right)/N$$

(10) $$\mathrm{RMSE}={\left(\left(\sum_{i=1}^{N}{\left|f_{\mathrm{Ref}}\left(i\right)-f_{\mathrm{FBP}}\left(i\right)\right|}^{2}\right)/N\right)}^{1/2}$$

(11) $$\mathrm{NMAD}=\left(\sum_{i=1}^{N}\left|f_{\mathrm{Ref}}\left(i\right)-f_{FBP}\left(i\right)\right|\right)/\left(\sum_{i=1}^{N}\left|f_{\mathrm{Ref}}\left(i\right)\right|\right)$$

where f and $\overset{\wedge }{f}$ represent the network output and label sinogram, $f_{FBP}$ and $f_{\mathrm{Ref}}$ represent the reconstructed image and ideal image, respectively, i represents the pixel index in the image, and N is the total number of pixels of the image. The closer the values of MAE, RMSE, and NMAD to 0, the smaller the differences between the ideal image and the network results.

### 3.2. Simulation Results

In the simulated experiment, we used a clinical dataset to establish the experimental dataset. The dataset comprised information on 12 patients; the information of 10 patients was used for network training, and that of the remaining 2 patients was used for testing. We obtained 30,000 pairs of training data. The training performed 10,000 rounds and required approximately 30 h. After the network training was complete, the individual image processing time was less than 1 s. With the same dataset and number of training epochs, we compared our model with the following models: LI [12], FCN [25], and U-net [26].

**Figure 5:**
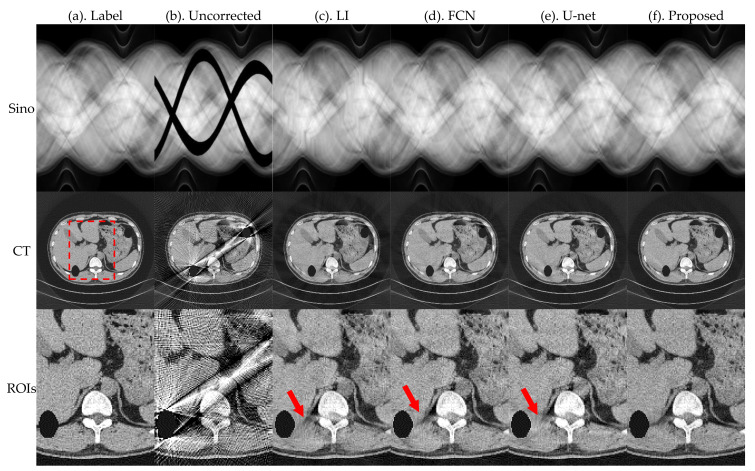
Simulation results of the pleural, where the first row is the sinogram, and the second row is the FBP-reconstructed results. The display window of sinogram is (0, 1). The display window of CT is (−0.1, 0.25).

**Figure 6:**
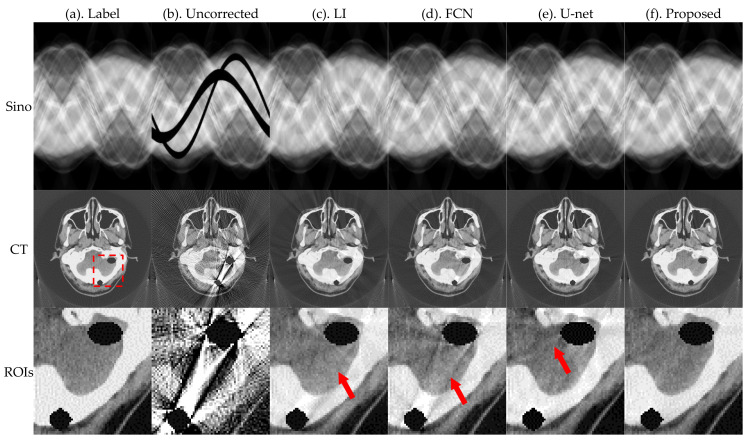
Simulation results of the cranial, where the first row is the sinogram, and the second row is the FBP-reconstructed results. The display window of sinogram is (0, 1). The display window of CT is (−0.1, 0.25).

Figure 5 and Figure 6 present the pleural and cranial results, respectively, obtained using the four algorithms. The first column shows the ground truth of the sinogram and the corresponding standard FBP-reconstructed results. We also show the results of the conventional LI model, deep-learning-based FCN, and U-net. Severe streak artifacts are observed in the CT images obtained by directly performing FBP reconstruction on the uncorrected sinogram in Figure 5 and Figure 6. Columns (c)–(f) in the second row show that different methods have different suppression effects on metal artifacts. The region of interests (ROI) marked by the red dashed box in the reconstructed image is enlarged in the third row. The enlarged ROIs show that our method reduces the global radial streak artifacts and generates images with more explicit boundaries and details.

To better evaluate the projection complement effect of each method, the MAE of each complement sinogram was calculated using Equation (9), and the results are presented in Table 1. RMSE and NMAD were used as metrics to accurately evaluate the artifacts correction effect of each network for the obtained reconstructed images and the selected ROI using Equations (10) and (11); the relevant results are presented in Table 2.

The MAE index values in Table 1 show that the proposed method has the best effect in repairing traces. FCN and U-net do not constrain the complement area; therefore, there are slight changes in the global pixel points, which result in large values of MAE. Corresponding evaluation metrics in Table 2 show that the RMSE and NMAD values of the corrected images obtained by the proposed method are further reduced relative to the other methods.

**Figure 7:**
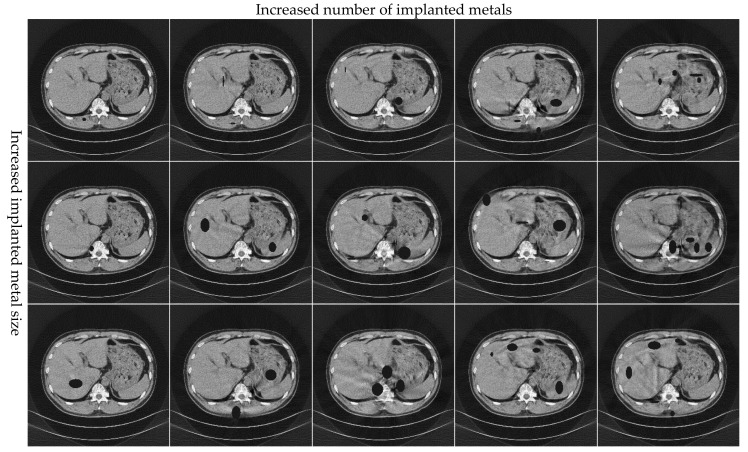
Simulation results of the pleural with different numbers, shapes, and sizes of metal implants. The display window of CT is (−0.1, 0.25).

Figure 7 presents the image after MAR processing of the pleural with different numbers, shapes, and sizes of metal implants. It shows that this method can effectively suppress metal artifacts with increasing metal size and number. However, when a large number of projection data are missing because of the presence of a metal object, it is challenging for the network to fully extract the features for image restoration, and it cannot achieve complete reconstruction.

### 3.3. Experimental Results

**Figure 8:**
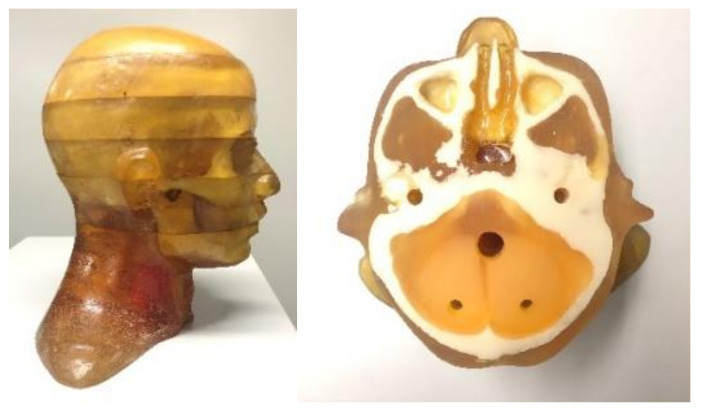
Real data experimental phantom: Chengdu dosimetric phantom.

To verify the application effect of this method for a realistic CT system, we performed a radiological anthropomorphic head phantom study for actual applications. The phantom is shown in Figure 8. The actual scanned CT system consisted of a micro-focus spot X-ray source (Hawkeye130, Thales, France), a flat-panel detector (Varian 4030E, USA), and a high-precision 4-axis linkage stage. To verify the adaptability of this network, we obtained the sinograms for different values of voltage and current. The X-ray tube voltage range was set to 90–120 kVp, and the current was set to 200–300 μA. We collected 31,000 pairs of sinogram images, of which, 30,000 were used for network training and 1000 for testing.

**Figure 9:**
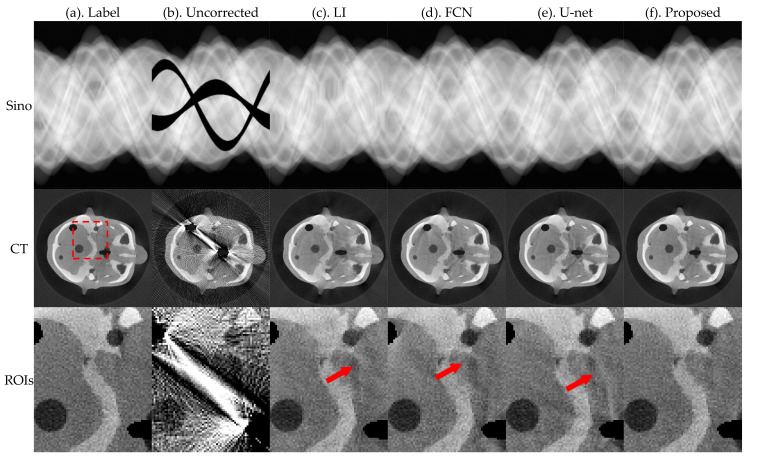
Results of head phantom, where the first row is the sinogram, the second row is the FBP-reconstructed results, and the third row is an enlarged view of the ROIs. The display window of sinogram is (0, 1). The display window of CT and ROIs is (−0.01, 0.025).

**Figure 10:**
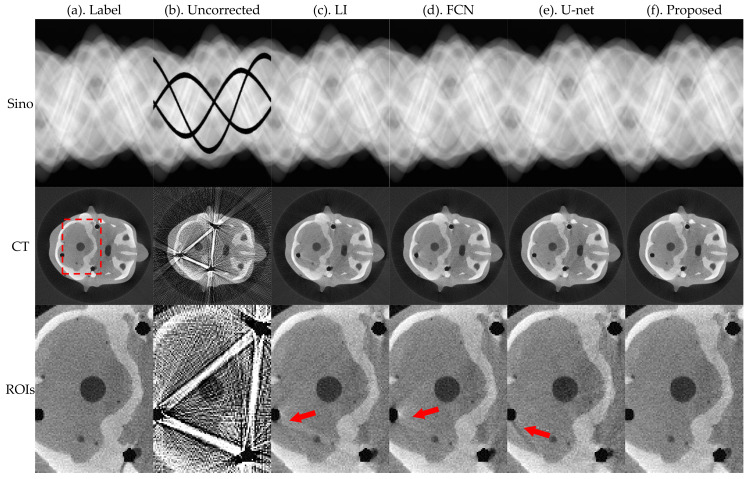
Results of head phantom, where the first row is the sinogram, the second row is the FBP reconstruction results, and the third row is an enlarged view of the ROIs. The display window of sinogram is (0, 1). The display window of CT and ROIs is (−0.01, 0.025).

The following results were obtained after 10,000 rounds of training. We used two sets of data in the actual network performance verification. The first set unplaced metal during the projection data acquisition process, and the second set contained three metal implants. To eliminate interference from other factors, the parameters and spatial position of the two sets were kept the same at the acquisition time. In the first set, we used simulated metal traces to create a test projection, and Figure 9 shows the related results. In the projection containing the metal, we directly set the metal-damaged traces to zero. Figure 10 shows the related results. With the same dataset and number of training epochs, we compared our model with the following models: LI [12], FCN [25], and U-net [26].

Figure 9 and Figure 10 present the results of the four algorithms. The first column shows the ground truth of the sinogram and the corresponding standard FBP-reconstructed results. The results show that our method can more accurately repair missing data of metal traces. Our method recovers detailed information of the metal artifacts in severely covered areas, eliminating the influence of metal artifacts. To further observe the details of the reconstructed images, we marked the ROIs with a red dashed box in Figure 9 and Figure 10; the ROIs are enlarged in the third row, and they show that our method reduces the global radial streak artifacts and generates images with more explicit boundaries and details. Our method achieves better removal of the radial streak artifacts.

To better evaluate the projection complement effect of each method, the MAE of each complement sinogram was calculated using Equation (9), and the results are presented in Table 3, which shows that the proposed method can accurately complement the missing metal trace regions. In the actual data experiment without metal placement, the MAE of the corrected results of the proposed method decreased by 60.055%, 55.850%, and 45.917%, respectively, compared with LI, FCN, and U-net. In the actual data experiment with metal placement, the MAE of the corrected results decreased by 59.963%, 53.504%, and 50.093%, respectively, compared with LI, FCN, and U-net. The MAE index shows that the proposed method can obtain better results in projection complement processing.

To accurately evaluate the artifact correction effect of each network, NMAD and RMSE were calculated for the obtained reconstructed images and the selected ROIs using Equations (10) and (11), respectively. NMAD and RMSE values in Table 4 show that in the actual experimental results, i.e., the reconstruction results obtained by applying the proposed method, the MAR effect was the best. In the results of projection image processing without metal placement, the NMAD index of the reconstructed image decreased by 50.286%, 36.029%, and 33.672%; the RMSE index value decreased by 48.214%, 38.536%, and 35.163%; the NMAD index of the ROIs decreased by 50.362%, 46.128%, and 39.241%; the RMSE index value decreased by 43.443%, 44.162%, and 34.906%, relative to LI, FCN, and U-net, respectively. In the results of projection image processing with metal placed, the NMAD index of the reconstructed image decreased by 42.331%, 42.739%, and 35.931%; the RMSE index value decreased by 37.850%, 32.997%, and 33.166%; the NMAD index of the ROIs decreased by 39.692%, 42.551%, and 37.083%; the RMSE index value decreased by 40.797%, 39.381%, and 36.053%, respectively, compared with LI, FCN, and U-net.

Thus, our method achieves good completion and correction in both simulation and actual tests. The results show that for the complement repair task of the CT sinogram, adding the feature loss of sinogram domain images can improve the effectiveness of the projection complement and optimize the correction effect.

### 3.4. Ablation Study

**Figure 11:**
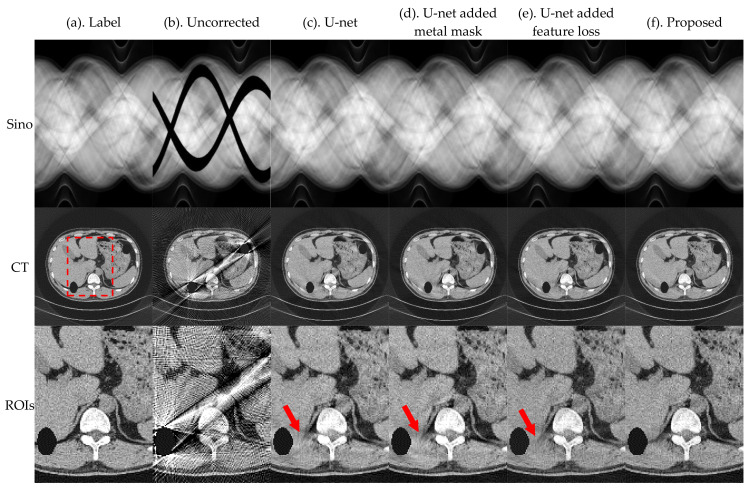
Simulation results of the pleural, where the first row is the sinogram, and the second row is the FBP reconstruction results. The display window of sinogram is (0, 1). The display window of CT is (−0.1, 0.25).

**Figure 12:**
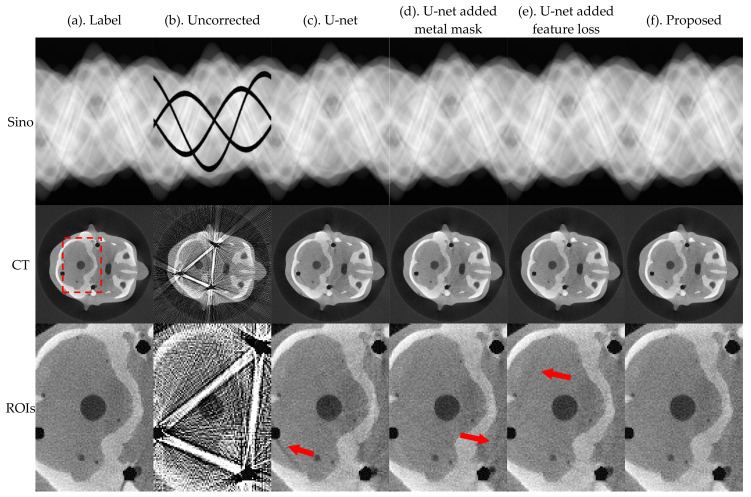
Actual results of head phantom, where the first row is the sinogram, the second row is the FBP reconstruction results, and the third row is an enlarged view of the ROIs. The display window of sinogram is (0, 1). The display window of CT and ROIs is (−0.01, 0.025).

In this section, we evaluate the effectiveness of the proposed method for metal traces masking and sinogram feature losses. Performance was evaluated based on the relevant results of the ablation experiment. Figure 11 and Figure 12 present the results of U-net, U-net-added metal mask, U-net-added feature loss, and the proposed method, for the pleural and the phantom head, respectively.

The image results in Figure 11 and Figure 12 and index evaluation results in Table 5 show that the proposed model can improve the effect of MAR. After integration, the correction effect is better than that when other modules are used independently.

## 4. Discussion

This study proposed a sinogram complementary deep learning method for MAR in CT images. The conventional MAR method requires an extended processing time due to multiple hyper-parameter settings. The proposed method can quickly complete the processing of a large volume of data.

In this method, collecting and processing datasets were the primary challenging tasks. By extracting the features of supplementary information from a large amount of training data, the repair effect and scope of application of the network can be improved. Based on the exceptional performance of metal artifacts in the sinogram domain, this method directly sets the metal-damaged traces to zero from the label image, which ensures matching of the image. A large amount of projection data were obtained by modifying the scanned tube voltage and current, which improved the diversity of training samples and further improved the applicability of the network.

In this study, we used a mask to limit the network patched area. In the sinogram, the area occluded by the metal projection was removed, and the projection information of other positions was accurate and effective. The features of these practical projection values can provide a reference for information completion in occluded areas and accurately reflect the original projection information. This method achieves efficient utilization of effective information by globally extracting features. The mask was used to constrain the repair area, and the nonlinear fitting ability of deep learning was used to realize the accurate repair of metal-damaged traces. We designed the sinogram feature loss based on the CT imaging principle and the consistency of the sinogram data distribution. The feature extraction ability of the network was improved through global constraints, ensuring the effectiveness of the supplementary data in the generated images.

## 5. Conclusions

In this study, network deepening of the network was used, which improved the feature extraction and image fitting ability of the network. We proposed a new complement method based on the image feature depth network in the sinogram domain. This method uses feature reuse, deconvolution, and feature connection to increase the deep feature extraction and expression capabilities of neural networks. The network corrects the sinogram image containing the metal artifact, and the output results can be directly reconstructed using the FBP algorithm. Effective and accurate supplementary data ensure the high quality of the reconstructed images. Visual observation and various relevant evaluation metrics of the reconstructed CT images show that the proposed method can effectively eliminate metal artifacts and significantly improve the quality of the reconstructed images.

The projection dataset under multiple voltages and currents can train a sinogram domain MAR network suitable for the CT system. The models of medical CT equipment are relatively uniform, the scanned patient parts are relatively fixed, and the imaging features are consistent. Therefore, this method will be more applicable to medical imaging problems.

Further research will focus on the sinogram optimization method based on deep learning for clinical CT imaging.

## Figures and Tables

**Table 1:** MAE results of complement sinogram of simulation experiments.

	(c). LI	(d). FCN	(e). U-Net	(f). Proposed
Pleural	28.2126	37.9773	40.6941	16.8108
Cranial	21.8383	22.0243	35.8966	11.9921

**Table 2:** RMSE and NMAD results of a reconstructed image in simulation experiments.

			(c). LI	(d). FCN	(e). U-Net	(f). Proposed
NMAD	Pleural	CT	0.1164	0.0995	0.0978	0.0724
		ROIs	0.1084	0.0930	0.0951	0.0699
	Cranial	CT	0.1216	0.1106	0.1066	0.0713
		ROIs	0.1150	0.1124	0.1064	0.0646
RMSE	Pleural	CT	8.2725	7.1038	7.0616	5.2356
		ROIs	12.6553	10.8224	11.4806	8.2140
	Cranial	CT	7.6252	6.3194	6.3905	4.3367
		ROIs	12.4006	12.0947	12.8702	7.5756

**Table 3:** MAE results of complement sinogram of actual experiments.

	(c). LI	(d). FCN	(e). U-Net	(f). Proposed
Case 1	0.9881	0.8940	0.7298	0.3947
Case 2	0.5385	0.4637	0.4320	0.2156

**Table 4:** NMAD and RMSE results of reconstructed image and ROIs of actual experiments.

			(c). LI	(d). FCN	(e). U-Net	(f). Proposed
NMAD	Case 1	CT	0.1050	0.0816	0.0787	0.0522
		ROIs	0.0967	0.0891	0.0790	0.0480
	Case 2	CT	0.0841	0.0847	0.0757	0.0485
		ROIs	0.0844	0.0886	0.0809	0.0509
RMSE	Case 1	CT	0.0616	0.0519	0.0492	0.0319
		ROIs	0.0854	0.0865	0.0742	0.0483
	Case 2	CT	0.0428	0.0397	0.0398	0.0266
		ROIs	0.0728	0.0711	0.0674	0.0431

**Table 5:** RMSE and NMAD results of reconstructed images from the simulation experiments.

			(c). U-Net	(d). U-Net Added Metal Mask	(e). U-Net Added Feature Loss	(f). Proposed
NMAD	Simulation results	CT	0.0978	0.0925	0.0859	0.0724
		ROIs	0.0951	0.0903	0.0838	0.0699
	Actual results	CT	0.0757	0.0586	0.0569	0.0485
		ROIs	0.0809	0.0589	0.0588	0.0509
RMSE	Simulation results	CT	7.0616	6.7823	6.1976	5.2356
		ROIs	11.4806	11.0051	9.8988	8.2140
	Actual results	CT	0.0397	0.0329	0.0330	0.0266
		ROIs	0.0674	0.0535	0.0520	0.0431

## Data Availability

The data and the code used for the manuscript are available for researchers on request from the corresponding author.
